# Nursing Management Experience of Acute Skin Failure in Critically Ill Patients: A Qualitative Study

**DOI:** 10.1002/nop2.70336

**Published:** 2025-10-17

**Authors:** QianSheng Jin, QiaoPing Chen, Gang Wu, YueFang Gao, WenTing Lu, Yan Sun, QingJie Zhu

**Affiliations:** ^1^ Intensive Care Unit Northern Jiangsu People's Hospital Yangzhou China; ^2^ Cardiac Intensive Care Unit Northern Jiangsu People's Hospital Yangzhou China; ^3^ Nursing Department Northern Jiangsu People's Hospital Yangzhou China

**Keywords:** critical illness, nursing, qualitative research, skin failure

## Abstract

**Aim:**

To explore the management experience of acute skin failure (ASF) in critically ill patients from the perspective of nurses and provide references for improving clinical practice.

**Design:**

A qualitative descriptive study.

**Methods:**

Using purposive sampling, semi‐structured interviews were conducted with 13 ICU nurses in a tertiary hospital in East City, China, between January and March 2025. Data were analysed using Colaizzi's phenomenological approach.

**Results:**

Four main themes emerged: (i) Cognitive dilemmas; (ii) Disease control and risk identification; (iii) Management optimisation‐from admission preparation to communication and (iv) Multidimensional exploration of nursing interventions. Nurses face challenges such as insufficient awareness, limited tools for early warning, fragmented communication and inadequate multidisciplinary collaboration. These findings suggest the urgent need for structured training, proactive care protocols and enhanced interdisciplinary coordination.

**Conclusion:**

Effective ASF management requires institutional support to build a standardised, technology‐enabled care model. Key priorities include tiered training programs for nurses, clearer interdisciplinary roles and the development of validated digital tools for assessment, documentation and real‐time risk monitoring. Policymakers and hospital administrators should take concrete actions to strengthen ASF nursing capabilities and integrate systematic response mechanisms into routine care.

**Implications for the Profession:**

This study highlights the importance of empowering nurses through structured education, protocol development and digital innovation. Promoting a culture of proactive identification and collaborative care may improve early intervention and reduce adverse skin outcomes. Nursing professionals are encouraged to lead in the integration of ASF management into quality improvement and safety initiatives.

**Patient or Public Contribution:**

No patient or public contribution.

## 1 Introduction

With advances in hemodynamic theory, peripheral circulation has become a critical focus in the management of critically ill patients (Levine and Delmore 2024), reflecting the trend toward more refined and precise clinical nursing care. The skin, as the largest organ of the body, accounts for 10%–15% of total body weight and receives 25%–33% of cardiac output (Chen et al. 2021), making it highly dependent on adequate blood perfusion similar to other vital organs. Acute Skin Failure (ASF) is defined as necrosis of the skin and underlying tissues caused by impaired perfusion during acute or critical illness, typically accompanied by hemodynamic instability and decreased tissue tolerance (Langemo and Parish 2022). The pathogenesis of ASF is complex and multifactorial. Currently, it is generally accepted that ASF primarily involves impaired tissue perfusion, dysregulated inflammatory responses, decreased tissue tolerance and disruption of the skin's barrier function. These interconnected mechanisms collectively contribute to the development and progression of ASF (Olshansky 2025).

Studies indicate that ASF carries a mortality rate as high as 42%, predominantly affecting areas such as the buttocks and sacrococcygeal region‐sites commonly associated with pressure injuries (PI)‐which complicates clinical management (Yang et al. 2025). Due to clinical similarities with pressure injuries, many ASF cases are mistakenly treated as pressure ulcers, which hinders the management of the primary illness and increases clinical complexity (Dalgleish et al. 2020). As an emerging and significant challenge in skin care, ASF has a profound impact on patient outcomes, making it an increasingly important focus in critical care nursing.

ASF may occur continuously in the Intensive Care Unit (ICU) setting, and nurses play a pivotal role in its prevention, identification and management. Especially in high‐risk environments like the ICU, nurses are often the first to observe skin changes, assess tissue perfusion, implement preventive interventions and participate in collaborative treatment (Zajac et al. 2024). Existing studies have preliminarily explored risk factors for ASF, including hypoperfusion, organ dysfunction, chronic comorbidities and malnutrition (Dalgleish et al. 2020). Researchers have also attempted to improve ASF recognition by developing tools to distinguish ASF from pressure injuries (Hill and Petersen 2020), constructing risk prediction models to identify high‐risk patients (Zhu et al. 2024) and combining indicators such as mottling scores and lactate levels to predict ASF in patients with septic shock (Jing et al. 2023). While these studies have significantly advanced our understanding of ASF risk factors and progression, how ASF should be managed in clinical practice remains underexplored. Moreover, limited attention has been given to nurses' subjective experiences, coping strategies and role perceptions when confronting ASF in real‐world settings. The lack of in‐depth qualitative research restricts our ability to uncover the practical challenges and experiential knowledge embedded in nursing care, thereby hindering the development of actionable, context‐sensitive and person‐centered strategies.

To this end, this study adopts a phenomenological qualitative approach to deeply explore the authentic experiences of ICU nurses in managing ASF, aiming to provide comprehensive theoretical support for developing more scientific, evidence‐based and clinically relevant nursing strategies, thereby promoting continuous improvement and optimization of critical care nursing practice.

## 2 Participants and Methods

### 2.1 Participants

This study employed a purposive sampling method to select ICU nurses in a tertiary hospital in East City, China from January to March 2025.

#### 2.1.1 Inclusion and Exclusion Criteria

##### 2.1.1.1 Inclusion Criteria

(1) Provided care for at least 20 patients with ASF; (2) Had at least three years of ICU work experience; (3) Held a valid nursing practice licence.

##### 2.1.1.2 Exclusion Criteria

(1) Nursing administrators, as they do not directly participate in frontline management, lead to limited practical experience related to ASF nursing care. (2) Nurses on hospital rotations or in advanced training programs do not independently undertake nursing tasks, resulting in insufficient hands‐on experience.

#### 2.1.2 Sample Size

This study was approved by the hospital ethics committee, and all participants provided informed consent and voluntarily participated in the study. The sample size was determined based on the principle of data saturation, meaning that data collection ceased when no new themes or perspectives emerged. To ensure the richness and depth of the data, nurses were recruited from a variety of ICU settings, covering diverse clinical experiences and challenges in managing ASF patients. Given that ASF presentations and nursing approaches may vary depending on patient populations and ICU specialties, including nurses from multiple units enhances the comprehensiveness and transferability of the findings. A total of 13 nurses were interviewed, including 7 from the general ICU, 1 wound and ostomy specialist nurse and 5 from various specialty ICUs (Burn ICU, Emergency ICU, Respiratory ICU, Neurosurgical ICU and Cardiovascular ICU). Participants were numbered 1 to 13 in the order of the interviews. Detailed participant information is presented in Table 1.

**TABLE 1:** General information of research objects.

Number	Age (years)	Education	Professional title	Working years	Working departments
N1	40	Master	Co‐chief superintendent nurse	19	General ICU
N2	48	Master	Chief superintendent nurse	24	Wound and ostomy clinic
N3	39	Undergraduate	Co‐chief superintendent nurse	18	general ICU
N4	32	Master	Supervisor nurse	16	General ICU
N5	42	Master	Supervisor nurse	21	General ICU
N6	38	Master	Co‐chief superintendent nurse	17	Burn ICU
N7	34	Master	Supervisor nurse	13	Emergency ICU
N8	36	Undergraduate	Supervisor nurse	15	Respiratory ICU
N9	38	Undergraduate	Supervisor nurse	17	Neurosurgical ICU
N10	36	Undergraduate	Supervisor nurse	15	Cardiovascular ICU
N11	39	Undergraduate	Supervisor nurse	18	General ICU
N12	40	Undergraduate	supervisor nurse	19	General ICU
N13	42	Undergraduate	Supervisor nurse	20	General ICU

### 2.2 Research Methods

#### 2.2.1 Interview Outline Development

Based on phenomenological research methods (Li and Liu 2018), the interview outline was initially developed through literature analysis and aligned with the study objectives. After conducting preliminary interviews with two general ICU nurses, the outline was revised and finalised to better elicit in‐depth responses. The final questions included: (1) What are your thoughts or feelings when managing patients with ASF? Can you describe a specific clinical situation where you dealt with ASF? (2) What challenges have you encountered in the process of managing ASF (e.g., assessment, intervention, communication with team members)? How did you address or overcome them? (3) From your experience, what nursing strategies or practices have been most effective in managing ASF? (4) What do you think can be improved in ASF management in the future (e.g., training, resources, interdisciplinary collaboration)? (5) Do you have any additional suggestions or recommendations regarding ASF prevention or care?

#### 2.2.2 Data Collection Methods

Using phenomenological research Li and Liu (2018), semi‐structured in‐depth interviews were conducted to collect data. The researcher conducted face‐to‐face interviews with 13 ICU nurses according to the interview outline. The interviews took place in the ICU teaching room to ensure a quiet and undisturbed environment. Before the interview, the researcher introduced the purpose of the study, explained the confidentiality of the data and obtained consent from the participants. The entire interview was recorded, and informed consent was signed. The interview duration was controlled between 30 and 60 min. During the interview, the researcher closely observed the non‐verbal expressions of the participants, adjusted the questioning strategy based on the participants' specific answers and encouraged them to express their true thoughts to obtain in‐depth information.

#### 2.2.3 Data Analysis Methods

Colaizzi's seven‐step method (Li and Liu 2018) was used for data analysis. This method offers a clear and systematic process to rigorously extract and organise core themes from participants' experiences. It emphasises preserving the original meaning of participants' statements and includes a critical validation step by returning findings to participants for confirmation, thereby enhancing the credibility and accuracy of the study. This approach ensures an authentic reflection of ICU nurses' experiences in managing ASF patients. The specific steps include: (1) Two researchers independently transcribed and proofread the audio recordings within 24 h after the interview; (2) Carefully reading transcripts to extract significant statements; (3) Open coding of repeated viewpoints; (4) Summarising codes to identify preliminary themes; (5) Providing detailed descriptions and constructing a thematic framework; (6) Analysing logical relationships among themes; (7) Returning findings to some participants for member checking. Two researchers independently analysed the data, repeatedly compared results and reached consensus through discussion to ensure accuracy and consistency. Two researchers independently conducted data analysis and repeatedly compared and verified the results. In case of disagreement, the research team discussed the matter and reached a consensus to ensure the accuracy and consistency of the analysis.

### 2.3 Ethical Considerations

This study was approved by the Ethics Committee of a tertiary general hospital in East City, China (Ethics Approval No.: 2025ky128). The research strictly adhered to ethical principles outlined in the Declaration of Helsinki (World Medical Association 2013). Prior to the interviews, participants were fully informed about the study's purpose and procedures. Participation was entirely voluntary, and participants retained the right to withdraw at any point without providing a reason. All textual and audio data were anonymised and kept strictly confidential to protect participants' privacy.

## 3 Result

### 3.1 Theme One: Disease Awareness

#### 3.1.1 Insufficient Attention: A Neglected Clinical Problem

Nurses widely reported that ASF is insufficiently recognised and often misclassified as PI in clinical settings, lacking an independent mechanism for incident reporting and management. In addition, early warning tools for ASF have not yet been promoted in clinical practice. Nurses often lack awareness of dynamic risk assessment, leading to retrospective identification only after skin damage has occurred‐missing the optimal window for intervention. Furthermore, many nurses still perceive ASF merely as a natural outcome of disease progression, which undermines their confidence in proactive nursing interventions and reduces the initiative and systematisation of care efforts.

N4: ‘*Whether ASF has occurred is sometimes hard for us to argue definitively. For instance, patients with multiple organ failure (MSOF) often present with skin breakdown that is clearly a result of ASF, yet nurses still have to spend a lot of time reporting it as an adverse event.’ The* lack of a dedicated reporting mechanism for ASF leads to its passive inclusion under the PI category in clinical practice, which not only wastes resources but also dampens nurses' motivation for identifying and reporting ASF accurately.

N6: ‘*ASF is often noticed only in hindsight. The patient develops a PI, and then we start to wonder‐‘we've already implemented protective measures, so why is there still a breakdown?’ Circulation has been poor all along‐maybe it was ASF.’ ASF* is often retrospectively identified by nurses only after skin damage becomes apparent. While this reflection may help in summarising clinical management experience, it also reveals an absence of robust early warning systems and a lack of awareness regarding continuous risk assessment.

N8: ‘*ASF is just a natural phenomenon in disease progression and aging. There's not much we can do in terms of nursing care.’ ASF* is often not regarded as a distinct clinical condition requiring intervention, but rather as a superficial manifestation of disease deterioration. This limited understanding reflects a lack of comprehensive cognition toward ASF among nurses.

#### 3.1.2 Professional Dilemma: The Ambiguity in Definition and Management

Nurses face both cognitive and practical challenges in the management of ASF. The absence of unified definitions and standardised management guidelines leads to blind spots in clinical recognition and timely response. Nurses often have limited autonomy in initiating interventions, making it difficult to deliver prompt and effective care. Current nursing protocols are largely extrapolated from PI management experiences, with few ASF‐specific guidelines available. This knowledge gap contributes to the clinical difficulties and sense of helplessness nurses encounter during practice.

N3: ‘ASF *feels new‐new in the sense that most nurses still don't know how to determine when ASF is occurring, and its prevention and treatment are quite tricky. But ASF also feels old‐old in that it resembles other forms of organ failure, where aggressive treatment of the primary disease may be the only viable option.’ This* statement reflects a paradox in nurses' understanding of ASF: on one hand, as a relatively new concept, ASF lacks mature criteria for clinical identification and intervention, making it challenging to manage; on the other hand, nurses often view ASF as an inevitable result of disease progression, believing that addressing the primary disease is the only meaningful intervention. These uncertainties leave nurses feeling lost and unsupported in their clinical decision‐making.

N5: ‘*It's not scientific to rely solely on nurses to manage ASF*. *We don't have prescribing authority, and we can't decide on treatment or medications.’ Nurses* emphasise that the management of ASF should not fall solely on the nursing profession. Due to a lack of independent prescribing authority, nurses are restricted to executing medical orders, which limits their ability to intervene autonomously. As a result, initiating interdisciplinary collaboration for ASF management becomes difficult in practice.

N11: ‘*When it comes to PI, we're all familiar with prevention and treatment. But if it's ASF, the interventions I know are still the same ones.’ Due* to the scarcity of targeted training and clinical guidelines, nurses often apply PI‐related approaches directly to ASF cases. This lack of condition‐specific strategies leads to a profound sense of powerlessness among nurses, further compromising the specificity and quality of care provided.

#### 3.1.3 Cognitive Pressure: A Challenging Task Without a Clear Path

ICU nurses commonly face substantial cognitive pressure and systemic constraints in managing ASF. Their current knowledge base and proficiency in implementing effective interventions are often inadequate, resulting in a strong sense of powerlessness during clinical practice. Moreover, the blurred boundary between ASF and PI, along with unclear responsibility attribution, places an additional psychological burden solely on nurses, which undermines their motivation and compromises the quality of care. These issues reveal a lack of adequate knowledge support and institutional safeguards in ASF management, underscoring the need for urgent improvements.

N2: ‘*Managing patients with ASF is extremely stressful. Even senior nurses feel that our existing knowledge and nursing measures can't really prevent it from happening. [Sighs]’ Nurses* often experience anxiety related to their limited capacity to manage ASF effectively. When existing knowledge and interventions fail to offer solutions, feelings of helplessness become even more pronounced.

N7: ‘Every *time I take care of this kind of patient, I'm on edge. I don't know what the final ‘straw’ will be that breaks the skin. I just don't want it to happen on my shift.’ The* unpredictability of ASF occurrence intensifies nurses' anxiety. In the absence of clear early warning protocols or management strategies, their sense of psychological safety remains unfulfilled.

N9: ‘ASF *is still reported as a PI in the quality monitoring system. In other words, it's like managing a ticking time bomb of a reportable event‐I'm always prepared to take the blame.’ In* current quality monitoring systems, ASF and PI are not differentiated. Nurses, rather than being supported as clinical caregivers, are often held responsible for adverse outcomes‐an obviously unfair and stressful burden.

N10: ‘*ASF feels like a ‘burden’‐who's responsible? Who's in charge of managing it? If these questions aren't clear, it's definitely detrimental to the patient.’ Clinical* nurses are often the policy implementers within hospital systems. When roles and responsibilities are undefined, accountability becomes blurred. Nursing managers and administrators should address these ambiguities by establishing clear protocols, thereby ensuring both care quality and patient safety.

### 3.2 Theme Two: Disease Control and Risk Identification

#### 3.2.1 Focusing on the Root Cause: Primary Disease Treatment Is Key

Nurses place great emphasis on treating the primary disease and proactively communicate risk information to physicians through written records, imaging data and other means. However, communication largely depends on individual initiative and lacks standardised procedures. Moreover, physicians cannot monitor patients around the clock, which may affect the timeliness of information transmission, hindering early identification and intervention. There is an urgent need to establish systematic communication mechanisms to improve collaboration efficiency and patient safety.

N2: ‘*ASF ultimately results from inadequate perfusion. Once microcirculation improves, the problem of skin failure in patients will naturally be resolved.’ Nurses* generally agree that microcirculatory perfusion status is closely related to the occurrence of ASF and therefore emphasise prioritising control of the primary disease to ensure adequate blood flow. However, this ‘treat the root cause’ mindset may overlook the dynamic monitoring and timely intervention of skin condition during disease progression, causing skin issues to emerge passively in the later stages.

N7: ‘I *like to write down the high‐risk factors I identify one by one on a piece of paper. Besides, I note the duration of each high‐risk factor and take videos and photos for comparison, which helps unify communication with doctors.’ By* meticulously recording high‐risk factors and their duration, and supplementing with multimedia materials such as videos and photos, nurses enhance the clarity and accuracy of risk information. This approach reflects nurses' proactivity and professionalism in risk communication, facilitating physicians' comprehensive understanding of patient status and promoting effective communication and collaboration.

N8: ‘Sometimes *patients require large doses of vasoactive drugs to maintain target blood pressure, and their distal limbs often show cyanosis. The insufficient microcirculatory perfusion may persist for a long time, but the patient's disease is continuously progressing, and doctors cannot adjust drug dosages bedside 24/7.’ In* ongoing care, nurses must closely observe subtle changes in circulatory perfusion. When patient conditions rapidly evolve and physicians cannot be present at all times, nurses often serve as critical intermediaries bridging clinical changes and treatment responses. However, due to the absence of systematic communication mechanisms, nurses' judgements and feedback on potential risks may not receive timely physician responses, increasing the risk of skin complications.

#### 3.2.2 Early Warning Mechanism: Identification and Response to High‐Risk Factors

Nurses possess a certain level of ability to identify ASF high‐risk factors and demonstrate proactive communication awareness. However, there is a lack of standardised reporting pathways in clinical practice, which may result in information omission and delays. Early signs of ASF are often subtle, increasing the difficulty of timely recognition. Some departments currently use paper‐based forms for dynamic assessment and are actively exploring digital tools to enhance communication efficiency and standardise risk prevention.

N3: ‘*Risk factors such as skin perfusion status and skin temperature are easy to understand, and we are very proactive in communicating with doctors. The problem is that we may not convey everything, and doctors don't always have time to listen.’ Nurses* are sensitive to identifying high‐risk factors for ASF and are willing to communicate actively. However, clinical communication often relies on individual experience and judgment, lacking standardised reporting channels. This can lead to information omission and untimely physician response, which adversely affects the accuracy and timeliness of nursing interventions.

N6: ‘Apart *from mottled skin, early signs of ASF are not obvious, but once skin breakdown occurs, recovery becomes very difficult.’ Nurses* generally agree that clinical manifestations of ASF are not prominent, and the intervention window is often missed by the time skin damage is visible. Nursing plans need to be anticipatory and supported by more precise identification tools.

N13:‘Our department uses a self‐developed paper form for ASF risk factor assessment every shift. We are now planning to incorporate it into an electronic system, which will facilitate nurses*’ dynamic assessments and make it easier for doctors to review.’ Departments* have implemented paper‐based ASF high‐risk factor assessment forms for shift handovers and dynamic evaluation and are actively promoting digital workflow construction to improve the timeliness of risk identification and the structural communication between medical and nursing staff.

### 3.3 Theme Three: Management Optimization‐From Admission Preparation to Communication

#### 3.3.1 Proactive Nursing: Preparatory Strategies Upon ICU Admission

Preparatory measures before and during the early phase of ICU admission facilitate the establishment of standardised protocols for admitting patients with ASF. Clear preparation of materials and targeted care for high‐risk areas promote systematic and standardised nursing practice.

N1: ‘*When admitting shock patients, I prepare warming devices such as heating blankets in advance. If a patient's skin temperature is low or mottled before warming, the effect will definitely be compromised.’ Early* preventive nursing upon ICU admission is crucial for ASF prevention. Early intervention helps avoid passive reactive care later in the patient's stay.

N5: ‘*For patients admitted due to poor circulation, I routinely prepare water bags and pressure‐relief cushions.’ Targeted* preparation for high‐risk patients reflects the application of proactive nursing strategies, which enhance nursing efficiency and patient comfort.

N9: ‘For *patients with shock or sepsis, especially those with prolonged microcirculation disorders, I proactively apply protective ointment to the sacrococcygeal area and hips.’ Local* protective measures for specific high‐risk areas improve proactive protection awareness.

N12: ‘*Our unit is equipped with warming gloves and foot covers, which we always have ready at the bedside for patients at high risk of skin failure.’ Combining* unit resource allocation with nursing interventions provides hardware support for proactive nursing and enhances overall preventive capacity.

#### 3.3.2 Condition Reporting: How to Accurately Convey Key Information

Nurses focus on selecting key indicators during patient condition reporting, using clinical tools to enhance the accuracy and efficiency of information transmission, ensuring more precise and timely communication.

N6: ‘*Skin perfusion pressure and skin temperature are the key indicators I emphasise in my condition reports. They are easy to obtain and have clear clinical significance*.’Focusing on key and easily accessible indicators helps improve the efficiency and accuracy of information transmission, enabling physicians to make prompt judgements.

N10: ‘*Photos of the skin taken by the portable digital assistant (PDA), a handheld device equipped with wristband scanning and photo capture functions, are automatically uploaded with real‐time timestamps. Why don't we make good use of this?’ Encouraging* the use of PDA‐uploaded photos with timestamps enables dynamic, objective documentation, improving the completeness of condition reports and communication efficiency.

#### 3.3.3 Documentation and Handover: Ensuring Information Completeness and Continuity

The use of standardised and unified documentation tools improves information transmission efficiency, reduces nurses' workload and promotes nursing standardisation. Standardised processes ensure the completeness and continuity of ASF‐related information, guaranteeing the coherence of nursing care. The iterative upgrading of electronic systems will support dynamic ASF monitoring, enhance the timeliness and accuracy of records, facilitate multidisciplinary collaboration and optimise nursing management and clinical efficiency.

N5: ‘Previously, *ASF information was recorded sporadically. After switching to a unified skin observation record sheet, everyone knows exactly where to document, making it easier to find and track, thus reducing duplicated efforts.’ The* adoption of standardised documentation tools not only enhances information management efficiency but also alleviates nurses' workload and fosters systematic and standardised nursing practice.

N7: ‘The *skin observation record sheet clearly defines what and where to document, so information handovers during shift changes are more complete, reducing omissions*.’Standardised ASF documentation processes improve the integrity and continuity of information. During handovers, incoming nurses can quickly review whether ASF has occurred or its progression status, ensuring the continuity of care.

N11: ‘Our *new electronic information system is adding an ASF monitoring module for patients with hypovolemic shock. I'm really looking forward to it!’ Upgrades* to electronic information systems provide technical support for dynamic ASF monitoring and management, potentially improving the timeliness and accuracy of ASF response, facilitating multidisciplinary collaboration and enhancing nursing management quality and clinical teamwork efficiency.

#### 3.3.4 Communication With Patients' Families: Enhancing Understanding and Trust

ASF occurrence is often misunderstood by families as a result of nursing negligence, placing significant communication pressure on nurses. Due to the unique environment of the ICU, nurses are primarily responsible for information delivery. The lack of effective communication strategies can easily lead to nurse–patient–family conflicts. Clearly distinguishing ASF from PI, conducting proactive health education and establishing standardised communication and feedback mechanisms can improve families' understanding and trust, thereby safeguarding nursing quality and team collaboration.

N1: ‘Nurses *practically ‘turn pale’ when talking about skin issues‐we fear skin problems the most. Even when we have done everything right, sometimes it feels really frustrating.’ Nurses* often face significant psychological pressure due to ASF being misattributed as nursing errors. This reflects insufficient professional knowledge, education, and an underdeveloped nurse–patient–family communication system.

N4:‘The skin is the largest organ of the body. Families can accept failures of other organs but not skin failure. I believe this is due to inadequate communication.’ Family acceptance of ASF is lower compared to other organ failures. Nurses need to strengthen scientific explanations and guidance about ASF characteristics during condition communication to correct family misconceptions. Clearly documenting ASF education in communication records helps gain family understanding.

N8:‘Distinguishing between PI and ASF is not about shifting blame when skin problems occur, but indirectly shows families we have already made efforts.’ Clearly differentiating ASF from PI not only clarifies responsibilities but also conveys the professionalism and proactive nature of nursing work to families, helping reduce unnecessary disputes and misunderstandings.

N11: ‘Most of the time, ICU patients*’ families are not at the bedside. Patient conditions can only be communicated to families through us, and there is inevitably a delay in their understanding.’ The* absence of families at the bedside makes nurses the primary communicators. If communication is not timely, families remain unaware of the latest skin condition status, making it difficult for them to accept rapid deterioration.

### 3.4 Theme Four: Multidimensional Exploration

#### 3.4.1 Skin Warmth Management: Strengthening Multidisciplinary Responsibility

Multidisciplinary teams commonly overlook patient warming, resulting in a lack of systematic management in this critical aspect. Although nurses attempt to raise awareness by posting reminders, a unified management protocol has yet to be established. Temperature management urgently requires attention and joint efforts from the entire multidisciplinary team.

N2:‘Warming is not just the nurses' *responsibility. Do physicians pay attention during diagnosis and treatment? Do medical technicians consider it during examinations? Do rehabilitation therapists focus on it during rehabilitation care?’ Physicians prioritise* disease treatment implementation, medical technicians focus on examination procedures, and rehabilitation therapists emphasise the execution of rehabilitation activities. Consequently, multidisciplinary personnel tend to neglect the importance of warming measures. Since temperature is one of the five vital signs, it is unreasonable for nurses alone to bear the main responsibility.

N13: ‘*Warming is easily overlooked. I mark ‘Pay attention to warming’ on the patient's bedside reminder board and hang department‐made signs. This way, hopefully, no one will forget.’ Nurses* attempt to remind all staff through posting visual prompts, but these are mostly spontaneous actions without widespread consensus. The department lacks systematic and standardised protocols for patient temperature management.

#### 3.4.2 Nutritional Management: Enhancing Skin Repair Capacity

Nutritional management is a key factor in improving skin repair ability, but multidisciplinary collaboration mechanisms remain underdeveloped. Although nurses can detect signs of malnutrition, they often lack professional training and institutional support to effectively participate in nutritional assessment and intervention. Dietitians mainly contribute through consultations, making it difficult to ensure continuity in the dynamic adjustment of nutritional plans.

N1: ‘*Results of nutritional indicators like albumin are often reviewed and managed by physicians, with limited nurse involvement.’ Nurses'* limited expertise in interpreting lab indicators and providing nutritional guidance confines them mostly to implementation roles, hindering participation in nutritional decision‐making.

N7: ‘*When patients have poor gastric emptying or severe diarrhoea leading to malabsorption, we should consult a dietitian to discuss and adjust nutritional formulas or switch nutritional support routes.’ Malabsorption* is common clinically, but the lack of regular dietitian involvement and cross‐disciplinary support restricts the implementation of individualised nutritional therapy.

N12: ‘*We often say malnourished people look pale and dull, and this ‘complexion’ is also reflected in critically ill patients with dry and dull skin‐something nurses should observe and report.’ As* the primary observers of malnutrition symptoms, nurses are not involved in formulating patients' nutritional plans.

#### 3.4.3 Protective Measures: Challenges in Multilevel Intervention Strategies

Multilevel protective interventions face practical challenges. Firstly, skin care agents are difficult to use routinely for prevention due to insurance reimbursement restrictions and insufficient family awareness. Secondly, protective devices such as water bags are prone to displacement during use, reducing intervention effectiveness. Additionally, patients often lack proper understanding of activity limitations and appropriate bed mobility guidance, resulting in prolonged localised tissue pressure. The lack of standardised promotion and implementation of interventions highlights the need to strengthen patient education, family communication, improve insurance policies and promote systematic and standardised nursing interventions.

N3: ‘*Skin protectants like Cavilon, buttock creams, and pressure‐relieving cotton pads cannot be reimbursed by insurance and are relatively expensive. Some families feel preventive use is a waste.’ Economic* burdens and insurance policy limitations restrict the use of preventive skin care products, while inadequate family awareness affects cooperation, limiting the broad application of protective measures.

N9: ‘I *place water bags under patients every time I turn them, but they quickly shift again once the patient moves.’ Protective* devices used in bed are prone to displacement, compromising protection efficacy; thus, dynamic management and supervision during nursing care are necessary.

N10: ‘*Patients often ask, ‘Can I move a bit? I feel stiff all over.’ Especially those with indwelling catheters may mistakenly believe they must not move unless told otherwise. Patients'* insufficient understanding of activity restrictions and lack of proper mobility guidance lead to prolonged immobilisation and continuous local tissue pressure, increasing ASF risk.

#### 3.4.4 Multidisciplinary Collaboration: Building a Comprehensive Management System

Current ASF management suffers from unclear division of responsibilities and low team collaboration efficiency within multidisciplinary teams, lacking systematic and standardised management mechanisms. Both departmental and hospital levels need to establish clear cross‐professional teams and closed‐loop management systems, improve information sharing and communication platforms, strengthen the execution of multidisciplinary ‘help‐seeking’ mechanisms and enhance collaboration quality and management efficiency.

N4: ‘*Specialists should handle specialised tasks. Nurses are the main force in PI management and prevention. As for ASF, that needs further consideration.’ Some* nurses question role divisions in multidisciplinary collaboration, perceiving ASF management as lacking clear responsibility assignments, which hinders efficient teamwork.

N12: ‘Departments *should form ASF response teams comprising physicians, primary nurses, and PI specialist nurses. Who assesses, manages, and controls quality should all be clearly defined.’ There* is an urgent need for systematic multidisciplinary collaboration mechanisms at both department and hospital levels, with clear responsibility divisions and cross‐professional teams forming closed‐loop management.

N13: ‘*For complex skin cases, after evaluation by nurses and attending physicians, the skin status is referred* via *DingTalk platform to specialised departments such as burn or wound ostomy care. These departments provide recommendations or treatment plans, which the attending physician reviews and decides whether to adopt. Overall, we must know how to ‘seek help’. Information* sharing and interdepartmental collaboration platforms remain inadequate. Communication pathways and professional support channels among disciplines are insufficient, and the ‘help‐seeking’ mechanism is not well executed, affecting management efficiency.

## 4 Discussion

### 4.1 Correcting Cognitive Misunderstandings to Promote Nurses' Active Response to ASF

This study found that ICU nurses often display uncertainty and hesitation in managing ASF, due to a lack of conceptual clarity, unclear roles and insufficient clinical guidelines. These cognitive misunderstandings directly weaken nurses' sense of responsibility and confidence, reducing their initiative in early identification and intervention. For instance, nurses were unsure whether ASF fell under their scope of care, and often passively waited for physician orders‐highlighting how cognitive barriers hindered clinical action. To address these issues, institutions should implement targeted educational interventions. Foundational training on ASF‐related pathophysiology and risk factors can be integrated into orientation or routine continuing education. Advanced modules, such as case‐based simulations or interdisciplinary workshops, can be offered to senior and wound care specialist nurses. Pocket guides and flowcharts may also support bedside decision‐making in ambiguous cases.

Moreover, ASF still lacks standardised diagnostic criteria and is not included in international disease classification systems (Berlowitz and Levine 2025), which contributes to clinical ambiguity. The absence of clear perfusion‐based indicators makes it difficult for nurses to distinguish ASF from pressure injuries, and increases the likelihood of mislabeling skin failure as nursing negligence (Yang et al. 2025; Pott et al. 2023). Punitive cultures surrounding skin injuries further discourage proactive identification. Therefore, correcting cognitive misunderstandings is not only essential for improving knowledge and confidence but also critical to promoting early detection, timely intervention and reducing preventable complications. Shifting from blame to support‐by promoting risk‐based assessment frameworks and fostering a safe reporting culture‐can help improve patient outcomes and reduce misdiagnosis (Berlowitz and Levine 2025; Chen et al. 2024; Xu et al. 2022).

### 4.2 Integrating Validated Risk Assessment Tools to Improve ASF Prevention and Management

Our results indicate that ICU nurses emphasise controlling primary diseases‐such as correcting hypotension and improving microcirculation‐as the cornerstone of ASF management. However, the absence of standardised communication mechanisms and the reliance on individual initiative can hinder timely identification and intervention of at‐risk patients. To address this gap, it is essential to incorporate validated assessment tools into routine clinical nursing workflows. For instance, peripheral perfusion indicators‐such as skin perfusion pressure and capillary refill time‐have been shown to be reliable early predictors of ASF (Zhang et al. 2024; Xu et al. 2022). Zhu et al. (2024) developed a logistic regression model highlighting sepsis, vasopressors and hypoalbuminemia as significant predictors. Additionally, the Skin Failure Clinical Indicator Scale (SFCIS), with an accuracy of 83.7%, can help differentiate ASF from pressure injuries (Hill and Petersen 2020). Building on these findings, we suggest that clinical protocols incorporate the use of perfusion‐related parameters in routine vital sign monitoring, especially for high‐risk ICU patients. For example, integrating the peripheral perfusion index into early warning systems and using the SFCIS during skin assessments could enhance diagnostic accuracy. Moreover, training nurses in the interpretation and use of these tools‐supported by digital documentation platforms‐would standardise reporting and facilitate interdisciplinary communication. By embedding validated tools into daily workflows and strengthening nurses' risk recognition and reporting capabilities, early detection of ASF may be significantly improved. This, in turn, may reduce misdiagnosis, enable timely interventions and ultimately improve patient outcomes.

### 4.3 Promoting Standardised ASF Management Through Digital Integration

The results of this study reveal that ICU nurses encounter multiple challenges across various stages of ASF care, including inconsistent patient preparation upon ICU admission, fragmented documentation, incomplete handovers, and delays or omissions in communication. These gaps reduce the continuity and quality of care and can also lead to misunderstandings between families and healthcare providers (Zhao et al. 2024). A systematic and standardised management process is essential to address these challenges. Existing evidence supports managing skin failure as a clinical syndrome, emphasising early identification of risk and protective factors and implementing full‐cycle nursing interventions (Berlowitz and Levine 2025; Pittman et al. 2024). However, ASF care remains underdeveloped in practice and lacks high‐quality guidelines to support its standardisation (Zajac et al. 2024). To bridge this gap, digital systems and electronic health records (EHRs) offer practical tools for standardising ASF care. For instance, integrating digital assessment checklists and condition reporting templates into EHRs can ensure timely and complete documentation across shifts, as highlighted by nurses in this study. The incorporation of photo capture and real‐time uploading through handheld devices facilitates visual tracking of skin changes and improves communication accuracy.

Furthermore, digital platforms can support automated alerts for high‐risk patients, reminders for preventive measures, and centralised access to interdisciplinary input, promoting closed‐loop communication and coordinated decision‐making. These functions help ensure that risk identification, early warning, protective interventions and family education are delivered consistently throughout the care cycle. In conclusion, advancing ASF management requires not only the establishment of standardised care protocols but also their integration into digital workflows. This approach can enhance efficiency, ensure care continuity and support multidisciplinary collaboration, ultimately improving patient outcomes and nursing quality.

### 4.4 Multidisciplinary Collaborative Treatment Approaches Are Effective Strategies for Managing ASF Patients

The findings of this study highlight that adequate warming, nutritional support, pressure relief care and multidisciplinary collaboration are essential components in the effective management of ASF. ASF management builds on the foundation of pressure injury prevention, with increased focus on addressing microcirculatory impairment mechanisms, which is well supported by current research. Warming is effective because it promotes local blood circulation through vasodilation, improved microcirculation, sympathetic nervous system regulation and enhanced metabolism, thereby increasing tissue perfusion and preventing ASF (Grayson 1988). Nutritional deficiencies disrupt immune function, collagen synthesis and skin tensile strength, while dehydration impairs cellular metabolism and wound healing. Studies indicate that wound healing demands increased energy, proteins, zinc, vitamins A, C, E and amino acids such as arginine and glutamine (Saghaleini et al. 2018; Munoz and Posthauer 2022). Additionally, Mody et al. emphasised that local skin pressure remains a significant risk factor for ASF, underscoring that prevention of pressure injuries remains integral to ASF management (Mody et al. 2025). Roderman et al. formed a multidisciplinary team including nurses, nutritionists, radiologists, vascular technicians and wound care specialists, which effectively prevented and treated pressure injuries in 480 patients over two years (Roderman et al. 2024).

Despite these benefits, several barriers impede effective multidisciplinary collaboration, including communication breakdowns among team members, unclear role definitions, limited institutional support and insufficient staff training. To overcome these challenges, healthcare institutions should cultivate a culture of collaboration by implementing policies that encourage teamwork, providing regular interdisciplinary education and training and establishing clear protocols for communication and responsibility sharing among disciplines. Combining warming, nutritional support, pressure relief, and multidisciplinary collaboration constitutes practical and effective strategies in clinical nursing practice. These findings highlight the need for continued research to identify optimal approaches for managing ASF and enhancing patient care quality.

## 5 Limitation

All participants in this study were recruited from a single tertiary hospital in East City, China, encompassing nurses from various ICU subspecialties. Differences in clinical practice and nursing management across these ICU subspecialties may introduce potential biases, which could affect the generalizability of the findings. Therefore, caution is warranted when applying these results to other healthcare settings or ICU environments, and further studies are needed to validate the findings in diverse contexts.

## 6 Conclusion

For hospital administrators and policymakers, the following recommendations are proposed: (1) Implement systematic, tiered training programs tailored to different nursing roles and levels to enhance knowledge, skills, and a strong sense of responsibility in ASF care; (2) Establish multidisciplinary collaboration frameworks with clearly defined roles and responsibilities, and regularly evaluate their effectiveness to ensure continuous improvement in teamwork and communication; (3) Develop and standardise ASF management protocols and validated assessment tools, integrated with digital platforms to enable dynamic monitoring and data sharing; (4) Increase investment in nursing resources, including personnel, advanced technologies, medical equipment, and information systems, to create a supportive care environment; (5) Strengthen health information systems to facilitate real‐time monitoring, intelligent alerts, and closed‐loop feedback on nursing care processes through electronic medical records and data platforms. In summary, this study calls upon hospital leadership and health policymakers to prioritise ASF nursing management by adopting concrete, actionable strategies that advance nursing quality, ultimately improving clinical outcomes and quality of life for critically ill patients, and promoting the overall development of the healthcare system.

## Author Contributions

Jin Qiansheng, Zhu Qingjie, and Wu Gang were responsible for study design. Chen Qiaoping and Gao Yuefang conducted data collection and processing. Sun Yan and Lu Wenting participated in data verification. All authors contributed to the critical revision of the manuscript and approved the final version to be published.

## Ethics Statement

This study was approved and conducted in strict accordance with ethical principles to protect participants' privacy.

## Consent

Informed written consent was obtained from all participants prior to their involvement. The study followed the guidelines set forth in the Helsinki Declaration.

## Conflicts of Interest

The authors declare no conflicts of interest.

## Data Availability

The data that support the findings of this study are available on request from the corresponding author. The data are not publicly available due to privacy or ethical restrictions.

## References

[nop270336-bib-0001] Berlowitz, D. R. , and J. M. Levine . 2025. “The Evolving Case for Skin Failure‐Beyond Pressure Injury.” JAMA Internal Medicine 185, no. 4: 359. 10.1001/jamainternmed.2024.7461. 39804609

[nop270336-bib-0002] Chen, X. P. , X. Y. Gong , and L. F. Zhao . 2024. “Qualitative Research on ICU Nurses' Cognitive Experience of Acute Skin Failure.” Chinese Journal of Emergency and Critical Care Nursing 5, no. 3: 205–210. 10.3761/i.issn.2096-7446.2024.03.002.

[nop270336-bib-0003] Chen, X. P. , Y. W. Lao , Y. Zhang , X. Y. Gong , and Y. Y. Zhuang . 2021. “Research Progress on Acute Skin Failure in Critically Ill Patients.” Chinese Journal of Nursing 56, no. 3: 376–380. 10.3761/j.issn.0254-1769.2021.03.010.

[nop270336-bib-0004] Dalgleish, L. , J. Campbell , K. Finlayson , and F. Coyer . 2020. “Acute Skin Failure in the Critically Ill Adult Population: A Systematic Review.” Advances in Skin & Wound Care 33, no. 2: 76–83. 10.1097/01.ASW.0000617844.69248.92. 31972579

[nop270336-bib-0005] Grayson, J. 1988. “Responses of the Microcirculation to Hot and Cold Environments.” Pharmacology & Therapeutics 38, no. 2: 201–214. 10.1016/0163-7258(88)90097-6. 3054939

[nop270336-bib-0006] Hill, R. , and A. Petersen . 2020. “Skin Failure Clinical Indicator Scale: Proposal of a Tool for Distinguishing Skin Failure From a Pressure Injury.” Wounds: a Compendium of Clinical Research and Practice 32, no. 10: 272–278. 33370243

[nop270336-bib-0007] Jing, C. C. , S. J. Wang , C. Y. Han , and L. L. Wei . 2023. “Predictive Value of Skin Mettling Score Combined With Lactic Acid for Acute Skin Failure in Septic Shock Patients.” Nursing Research 37, no. 13: 2347–2351. 10.12102/j.issn.1009-6493.2023.13.010.

[nop270336-bib-0008] Langemo, D. , and L. C. Parish . 2022. “The Past, Present, and Future of Skin Failure.” Advances in Skin & Wound Care 35, no. 2: 81–83. 10.1097/01.ASW.0000803596.89726.6e. 35050915

[nop270336-bib-0009] Levine, J. M. , and B. Delmore . 2024. “Pressure Injuries and Skin Failure.” Clinics in Geriatric Medicine 40, no. 3: 385–395. 10.1016/j.cger.2023.12.006. 38960532

[nop270336-bib-0010] Li, Z. , and Y. Liu . 2018. Nursing Research Methods [M]. People's Medical Publishing House.

[nop270336-bib-0011] Mody, L. , E. Rittenberg , and S. K. Inouye . 2025. “Pressure Injuries and Skin Failure‐Pressure Still Matters.” JAMA Internal Medicine 10, no. 4: 360–361. 10.1001/jamainternmed.2024.7464. 39804608

[nop270336-bib-0012] Munoz, N. , and M. E. Posthauer . 2022. “Nutrition Strategies for Pressure Injury Management: Implementing the 2019 International Clinical Practice Guideline.” Nutrition in Clinical Practice: Official Publication of the American Society for Parenteral and Enteral Nutrition 37, no. 3: 567–582. 10.1002/ncp.10762. 34462964

[nop270336-bib-0013] Olshansky, K. 2025. “It Is Time for a Consensus on a Definition for Skin Failure.” Journal of Wound Care 34, no. Sup 5: S3–S4. 10.12968/jowc.2025.0087. 40358504

[nop270336-bib-0014] Pittman, J. , J. M. Black , A. de Jesus , and W. V. Padula . 2024. “The Standardized Pressure Injury Prevention Protocol (SPIPP) Checklist 2.0: Content Validation.” Journal of Advanced Nursing 80, no. 6: 2584–2591. 10.1111/jan.15964. 37994188

[nop270336-bib-0015] Pott, F. S. , M. J. Meier , J. G. D. Stocco , F. F. C. Petz , H. Roehrs , and P. K. Ziegelmann . 2023. “Pressure Injury Prevention Measures: Overview of Systematic Reviews.” Revista da Escola de Enfermagem da USP 57: e20230039. 10.1590/1980-220X-REEUSP-2023-0039en. PMC10742601 38133528

[nop270336-bib-0016] Roderman, N. , S. Wilcox , and A. Beal . 2024. “Effectively Addressing Hospital‐Acquired Pressure Injuries With a Multidisciplinary Approach.” HCA Healthcare Journal of Medicine 5, no. 5: 577–586. 10.36518/2689-0216.1922. 39524937 PMC11547285

[nop270336-bib-0017] Saghaleini, S. H. , K. Dehghan , and K. Shadvar . 2018. “Pressure Ulcer and Nutrition.” Indian Journal of Critical Care Medicine 22, no. 4: 283–289. 10.4103/ijccm.IJCCM_277_17. 29743767 PMC5930532

[nop270336-bib-0018] World Medical Association . 2013. “World Medical Association Declaration of Helsinki: Ethical Principles for Medical Research Involving Human Subjects.” Jama 310, no. 20: 2191–2194. 10.1001/jama.2013.281053. 24141714

[nop270336-bib-0019] Xu, H. , Y. Jin , and Q. Huang . 2022. “Differentiation and Assessment of Acute Skin Failure and Pressure Injury: A Literature Review.” Journal of Nursing 37, no. 3: 109–112. 10.3870/j.issn.1001-4152.2022.03.109.

[nop270336-bib-0020] Yang, X. , F. Yan , S. Xie , et al. 2025. “Acute Skin Failure Knowledge, Attitudes and Practices Amongst Intensive Care Unit Nurses in China: A Multicentre Cross‐Sectional Survey.” International Wound Journal 22, no. 1: e70099. 10.1111/iwj.70099. 39800335 PMC11725354

[nop270336-bib-0021] Zajac, K. K. , K. Schubauer , and R. Simman . 2024. “The Unavoidable Pressure Injury/Ulcer: A Review of Skin Failure in Critically Ill Patients.” Journal of Wound Care 33, no. Sup9: S18–S22. 10.12968/jowc.2024.0079. 39283887

[nop270336-bib-0022] Zhang, X. M. , Y. Wang , and J. L. Gao . 2024. “Research Progress on Risk Assessment and Risk Factors of Acute Skin Failure in Seriously Ill Patients.” Journal of Nursing Management 24, no. 11: 958–962. 10.3969/i.issn.1671-315x.2024.11.008.

[nop270336-bib-0023] Zhao, N. , L. Wang , and D. Zhang . 2024. “Evaluation of a Pressure Injury Risk Assessment Module Based on a Nursing Information System in an Intensive Care Unit.” Alternative Therapies in Health and Medicine 24: AT10352. 39316526

[nop270336-bib-0024] Zhu, L. H. , Y. F. Shen , Q. Ren , and J. Lin . 2024. “Construction of a Risk Prediction Model for the Occurrence of Acute Skin Failure in Critically Ill Patients: A Prospective Study.” Journal of Nursing Research: JNR 32, no. 4: e338. 10.1097/jnr.0000000000000627. 39046359

